# [Hydrogen bis­(1,2,4-triazole)] 1,2,4-triazolium bis­(3-carb­oxy-4-hy­droxy­benzene­sulfonate) 1,2,4-triazole disolvate

**DOI:** 10.1107/S1600536810008603

**Published:** 2010-07-17

**Authors:** Ming-qiang Qiu

**Affiliations:** a College of Chemistry, Central China Normal University, Wuhan 430079, People’s Republic of China

## Abstract

The title compound, C_2_H_4_N_3_^+^·[H(C_2_H_3_N_3_)_2_]^+^·2C_7_H_5_O_6_S^−^·2C_2_H_3_N_3_, consists of two types of 1,2,4-triazole monocation, one protonated at the 2-site lying across a twofold axis and the other protonated at the 4-site with the H atom disordered over a center of symmetry, a 5-sulfosalicylate anion and a neutral 1,2,4-triazole mol­ecule. The component ions are linked into a three-dimensional network by a combination of N—H⋯O, N—H⋯N, O—H⋯O, O—H⋯N, C—H⋯O and C—H⋯N hydrogen bonds. In addition, benzene–benzene π–π inter­actions of 3.942 (2) Å [inter­planar spacing = 3.390 (2) Å] and C—O⋯π (3.331 Å) inter­actions are observed.

## Related literature

For potential applications of co-crystals, see: Aakeröy *et al.* (2009 ▶); Chen *et al.* (2010 ▶); For co-crystals involved 5-sulfosaliyclic acid or triazole, see: Jin *et al.* (2006 ▶); Kiviniemi *et al.* (2000 ▶); Meng *et al.* (2007 ▶, 2008 ▶); Ye *et al.* (2008 ▶). 
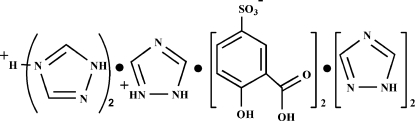


## Experimental

### 

#### Crystal data

C_2_H_4_N_3_^+^·C_4_H_7_N_6_^+^·2C_7_H_5_O_6_S^−^·2C_2_H_3_N_3_
*M* *_r_* = 781.73
Monoclinic, 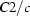

*a* = 21.2585 (5) Å
*b* = 5.1471 (2) Å
*c* = 32.2084 (15) Å
β = 106.669 (2)°
*V* = 3376.1 (2) Å^3^
*Z* = 4
Mo *K*α radiation
μ = 0.24 mm^−1^
*T* = 295 K
0.30 × 0.20 × 0.16 mm

#### Data collection

Bruker SMART APEX CCD area-detector diffractometer
Absorption correction: multi-scan (*SADABS*; Sheldrick, 1997 ▶) *T*_min_ = 0.921, *T*_max_ = 0.962
18315 measured reflections
3853 independent reflections
3005 reflections with *I* > 2σ(*I*)
*R*_int_ = 0.062

#### Refinement

*R*[*F* ^2^ > 2σ(*F* ^2^)] = 0.044
*wR*(*F* ^2^) = 0.124
*S* = 1.09
3853 reflections
240 parameters
1 restraint
H-atom parameters constrained
Δρ_max_ = 0.31 e Å^−3^
Δρ_min_ = −0.47 e Å^−3^

### 

Data collection: *SMART* (Bruker, 2001 ▶); cell refinement: *SAINT-Plus* (Bruker, 2001 ▶); data reduction: *SAINT-Plus*; program(s) used to solve structure: *SHELXS97* (Sheldrick, 2008 ▶); program(s) used to refine structure: *SHELXL97* (Sheldrick, 2008 ▶); molecular graphics: *PLATON* (Spek, 2009 ▶); software used to prepare material for publication: *PLATON*.

## Supplementary Material

Crystal structure: contains datablocks global, I. DOI: 10.1107/S1600536810008603/lh5006sup1.cif

Structure factors: contains datablocks I. DOI: 10.1107/S1600536810008603/lh5006Isup2.hkl

Additional supplementary materials:  crystallographic information; 3D view; checkCIF report

## Figures and Tables

**Table 1:** Hydrogen-bond geometry (Å, °)

*D*—H⋯*A*	*D*—H	H⋯*A*	*D*⋯*A*	*D*—H⋯*A*
N1—H1*A*⋯O4^i^	0.86	2.17	2.9231 (18)	145
N1—H1*A*⋯O5^ii^	0.86	2.46	2.984 (2)	120
N4—H4*A*⋯N2^iii^	0.86	2.09	2.931 (2)	166
N6—H6*A*⋯N6^iv^	0.86	1.81	2.667 (3)	175
N7—H7⋯O6	0.86	2.07	2.885 (2)	159
N7′—H7′⋯O5	0.86	2.50	3.145 (2)	133
N7′—H7′⋯O6^v^	0.86	2.12	2.8104 (19)	137
O3—H3*A*⋯O2	0.83	1.78	2.577 (2)	159
O1—H1⋯N3^v^	0.83	1.85	2.6791 (19)	178
C8—H8⋯O2^iii^	0.93	2.50	3.110 (2)	123
C9—H9⋯N5^vi^	0.93	2.62	3.381 (3)	139
C10—H10⋯O4^ii^	0.93	2.58	3.177 (2)	122
C10—H10⋯O5^ii^	0.93	2.47	3.278 (2)	145

Symmetry codes: (i) 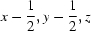; (ii) 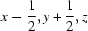; (iii) 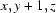; (iv) 
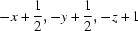
; (v) 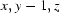; (vi) 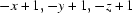.

**Fig. 1.**
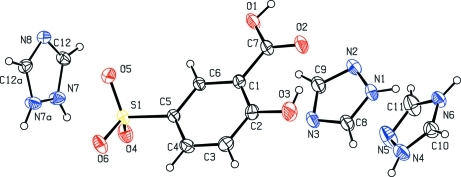
Molecular structures of (I), showing the atom-numbering scheme. Displacement ellipsoids are drawn at the 30% probability level and H atoms are shown as small spheres of arbitrary radii. Symmetry code: (a) 2 - x, y, 3/2 - z)

**Fig. 2.**
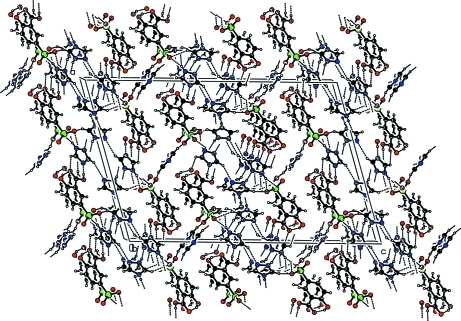
Part of the crystal structure of (I), showing the formation of the three-dimensional network. Hydrogen bonds are shown as dashed lines.

## supplementary crystallographic information

### Comment

Due to its potential applications in pharmaceuticals, the synthesis of co-crystals has become very attractive area of research recently (Chen *et al.*, 2010, Aakeröy *et al.*, 2009). Many cocrystals and organic salts were synthesized using 5-sulfosaliyclic acid and N-containing Lewis bases (Meng *et al.*, 2007, 2008). We here report our findings on the title compound I, *cf.* Scheme 1.

In compound (I), only the sulfonic-acid hydrogen atoms were transferred to triazole N atoms, resulting in a 5-sulfosalicylate anion and two type of cations *i.e.* one was protonated at 2- site lying across a twofold axis and the other protonated at 4-site with the hydrogen being disordered over a center of symmetry. Besides above mentioned, there is still one neutral 1,2,4-triazole molecule in (I) (Fig. 1). The N7—N7^v^ (2 - *x*, *y*, 3/2 - *z*) bond length of 1.309 (3)Å is apparently shorter than some analogs which should be largely attributed to its protonated position at the 2- site, but not the generally observed 4-site (Jin *et al.*, 2006; Ye *et al.*, 2008; Kiviniemi *et al.*, 2000).

In the packing structure of (I), the ionic components are linked into three-dimensional networks by a combination of N—H···O, O—H···O and C—H···O hydrogen bonds (Table 1 and Fig. 2). Analysis using *PLATON* (Spek, 2009) indicates that π···π interactions exist between symmetry-related benzene rings in these layers [centroid-to-centroid separation = 3.942 (2) Å, inter-planar spacing = 3.390 (2) Å and symmetry codes: 1/2 - *x*, 3/2 - *y*, 1 - *z*]. Additionally, the crystal structure was further consolidated by a O—H···π interaction which was scarcely observed [O3···*Cg*2 = 3.329 (2)\%A, *Cg*2 is the centroid defined by atoms N7/N8/C12 at (*x* - 1/2, *y* + 1/2, *z*) and atoms N7/N8/C12 at (-*x* + 3/2, *y* + 1/2, 3/2 - *z*)].

### Experimental

A 3:1 molar amount of 1,2,4-triazole (0.6 mmol, 41.4 mg) to 5-sulfosaliyclic acid dihydrate (0.2 mmol, 50.8 mg) were dissolved in 95% methanol (40 ml). The mixture was stirred for several minutes at ambient temperature and then filtered. The resulting colorless solution was kept in air for two weeks. Colorless block crystals of (I) suitable for X-ray diffraction were grown by slow evaporation at the bottom of the vessel.

### Refinement

H atoms bonded to aromatic C atoms were positioned geometrically with C–H = 0.93 Å, and refined in a riding mode [*U*_iso_(H) = 1.2*U*_eq_(aromatic C)]. H atoms bonded to N and O atoms were initially found in difference maps and then constrained to be at their ideal positions (N—H = 0.86Å and O—H = 0.82 Å). Their thermal factors were set k times of their carrier atoms (k=1.2 for N and 1.5 for O atoms, respectively). C12/N7' and N7/C12' atoms were occupationally disordered with occupancies of 0.5:0.5, respectively. H6A is disordered over a center of inversion and its occupancy was set 0.5.

### Crystal data

**Table:**

C_2_H_4_N_3_^+^·C_4_H_7_N_6_^+^·2C_7_H_5_O_6_S^−^·2C_2_H_3_N_3_	*F*(000) = 1616
*M**_r_* = 781.73	*D*_x_ = 1.538 Mg m^−^^3^
Monoclinic, *C*2/*c*	Mo *K*α radiation, λ = 0.71073 Å
Hall symbol: -C 2yc	Cell parameters from 6709 reflections
*a* = 21.2585 (5) Å	θ = 2.4–27.4°
*b* = 5.1471 (2) Å	µ = 0.24 mm^−^^1^
*c* = 32.2084 (15) Å	*T* = 295 K
β = 106.669 (2)°	Block, colorless
*V* = 3376.1 (2) Å^3^	0.30 × 0.20 × 0.16 mm
*Z* = 4	

### Data collection

**Table:**

Bruker SMART APEX CCD area-detector diffractometer	3853 independent reflections
Radiation source: fine focus sealed Siemens Mo tube	3005 reflections with *I* > 2σ(*I*)
graphite	*R*_int_ = 0.062
0.3° wide ω exposures scans	θ_max_ = 27.5°, θ_min_ = 2.0°
Absorption correction: multi-scan (*SADABS*; Sheldrick, 1997)	*h* = −27→27
*T*_min_ = 0.921, *T*_max_ = 0.962	*k* = −6→6
18315 measured reflections	*l* = −41→41

### Refinement

**Table:**

Refinement on *F*^2^	Primary atom site location: structure-invariant direct methods
Least-squares matrix: full	Secondary atom site location: difference Fourier map
*R*[*F*^2^ > 2σ(*F*^2^)] = 0.044	Hydrogen site location: inferred from neighbouring sites
*wR*(*F*^2^) = 0.124	H-atom parameters constrained
*S* = 1.09	*w* = 1/[σ^2^(*F*_o_^2^) + (0.0743*P*)^2^] where *P* = (*F*_o_^2^ + 2*F*_c_^2^)/3
3853 reflections	(Δ/σ)_max_ = 0.001
240 parameters	Δρ_max_ = 0.31 e Å^−^^3^
1 restraint	Δρ_min_ = −0.47 e Å^−^^3^

### Special details

**Table:**

Geometry. All e.s.d.'s (except the e.s.d. in the dihedral angle between two l.s. planes) are estimated using the full covariance matrix. The cell e.s.d.'s are taken into account individually in the estimation of e.s.d.'s in distances, angles and torsion angles; correlations between e.s.d.'s in cell parameters are only used when they are defined by crystal symmetry. An approximate (isotropic) treatment of cell e.s.d.'s is used for estimating e.s.d.'s involving l.s. planes.
Refinement. Refinement of *F*^2^ against ALL reflections. The weighted *R*-factor *wR* and goodness of fit *S* are based on *F*^2^, conventional *R*-factors *R* are based on *F*, with *F* set to zero for negative *F*^2^. The threshold expression of *F*^2^ > σ(*F*^2^) is used only for calculating *R*-factors(gt) *etc*. and is not relevant to the choice of reflections for refinement. *R*-factors based on *F*^2^ are statistically about twice as large as those based on *F*, and *R*- factors based on ALL data will be even larger.

### Fractional atomic coordinates and isotropic or equivalent isotropic displacement parameters (Å^2^)

**Table:**

	*x*	*y*	*z*	*U*_iso_*/*U*_eq_	Occ. (<1)
C1	0.66592 (8)	0.1594 (3)	0.66015 (5)	0.0349 (4)	
C2	0.66022 (9)	0.3282 (4)	0.69313 (6)	0.0412 (4)	
C3	0.70762 (9)	0.5162 (4)	0.70910 (6)	0.0450 (4)	
H3	0.7034	0.6283	0.7308	0.054*	
C4	0.76066 (9)	0.5383 (4)	0.69313 (5)	0.0397 (4)	
H4	0.7922	0.6655	0.7040	0.048*	
C5	0.76747 (8)	0.3715 (3)	0.66080 (5)	0.0308 (3)	
C6	0.72049 (7)	0.1834 (3)	0.64446 (5)	0.0326 (4)	
H6	0.7252	0.0719	0.6228	0.039*	
C7	0.61526 (8)	−0.0409 (4)	0.64341 (6)	0.0408 (4)	
C8	0.48133 (9)	0.4771 (4)	0.61003 (6)	0.0475 (5)	
H8	0.4816	0.5938	0.6321	0.057*	
C9	0.50432 (10)	0.2716 (4)	0.56064 (7)	0.0520 (5)	
H9	0.5260	0.2187	0.5408	0.062*	
C10	0.32077 (9)	0.5933 (4)	0.53084 (6)	0.0482 (5)	
H10	0.3107	0.6020	0.5571	0.058*	
C11	0.32417 (11)	0.4882 (5)	0.46875 (7)	0.0635 (6)	
H11	0.3153	0.4019	0.4423	0.076*	
C12	0.95952 (7)	−0.0581 (3)	0.72250 (5)	0.0361 (4)	0.50
H12	0.9251	−0.1117	0.6991	0.043*	0.50
N7'	0.95952 (7)	−0.0581 (3)	0.72250 (5)	0.0361 (4)	0.50
H7'	0.9277	−0.1077	0.7008	0.043*	0.50
S1	0.836787 (18)	0.39131 (8)	0.641015 (13)	0.03328 (15)	
N1	0.43718 (7)	0.2946 (3)	0.59598 (5)	0.0472 (4)	
H1A	0.4043	0.2664	0.6058	0.057*	
N2	0.45067 (8)	0.1586 (3)	0.56410 (6)	0.0513 (4)	
N3	0.52520 (7)	0.4707 (3)	0.58830 (5)	0.0472 (4)	
N4	0.36238 (8)	0.7445 (3)	0.51939 (6)	0.0538 (4)	
H4A	0.3849	0.8622	0.5361	0.065*	
N5	0.36563 (10)	0.6798 (4)	0.47949 (6)	0.0719 (6)	
N6	0.29550 (7)	0.4275 (3)	0.49953 (5)	0.0431 (4)	
H6A	0.2671	0.3075	0.4989	0.052*	0.50
N7	0.97459 (8)	0.1865 (3)	0.73276 (5)	0.0416 (4)	0.50
H7	0.9547	0.3209	0.7193	0.050*	0.50
C12'	0.97459 (8)	0.1865 (3)	0.73276 (5)	0.0416 (4)	0.50
H12'	0.9531	0.3318	0.7182	0.050*	0.50
N8	1.0000	−0.2185 (4)	0.7500	0.0355 (4)	
O1	0.62327 (6)	−0.1836 (3)	0.61157 (4)	0.0480 (3)	
H1	0.5922	−0.2874	0.6043	0.072*	
O2	0.56876 (6)	−0.0698 (3)	0.65856 (5)	0.0564 (4)	
O3	0.60993 (7)	0.3137 (3)	0.71072 (5)	0.0634 (4)	
H3A	0.5891	0.1885	0.6969	0.095*	
O4	0.81766 (6)	0.5286 (3)	0.60014 (4)	0.0488 (3)	
O5	0.85622 (6)	0.1272 (2)	0.63667 (4)	0.0494 (3)	
O6	0.88616 (6)	0.5346 (3)	0.67359 (4)	0.0527 (4)	

### Atomic displacement parameters (Å^2^)

**Table:**

	*U*^11^	*U*^22^	*U*^33^	*U*^12^	*U*^13^	*U*^23^
C1	0.0309 (8)	0.0349 (9)	0.0413 (9)	−0.0020 (7)	0.0143 (7)	0.0024 (7)
C2	0.0406 (9)	0.0445 (11)	0.0447 (10)	0.0000 (8)	0.0219 (8)	0.0003 (8)
C3	0.0496 (10)	0.0456 (11)	0.0445 (10)	−0.0010 (9)	0.0210 (8)	−0.0114 (8)
C4	0.0409 (9)	0.0370 (10)	0.0408 (9)	−0.0072 (8)	0.0112 (7)	−0.0053 (7)
C5	0.0293 (8)	0.0308 (9)	0.0321 (8)	−0.0024 (6)	0.0082 (6)	0.0014 (6)
C6	0.0312 (8)	0.0329 (9)	0.0363 (8)	−0.0044 (7)	0.0138 (7)	−0.0028 (7)
C7	0.0331 (9)	0.0409 (10)	0.0508 (10)	−0.0075 (8)	0.0158 (8)	0.0017 (8)
C8	0.0404 (10)	0.0487 (12)	0.0582 (12)	−0.0127 (9)	0.0217 (9)	−0.0063 (9)
C9	0.0432 (10)	0.0553 (12)	0.0634 (12)	−0.0136 (9)	0.0249 (9)	−0.0123 (10)
C10	0.0430 (10)	0.0528 (12)	0.0481 (11)	−0.0086 (9)	0.0121 (8)	−0.0094 (9)
C11	0.0566 (12)	0.0892 (17)	0.0477 (12)	−0.0238 (13)	0.0198 (10)	−0.0170 (11)
C12	0.0348 (8)	0.0347 (9)	0.0361 (8)	−0.0063 (6)	0.0059 (6)	−0.0029 (6)
N7'	0.0348 (8)	0.0347 (9)	0.0361 (8)	−0.0063 (6)	0.0059 (6)	−0.0029 (6)
S1	0.0259 (2)	0.0380 (3)	0.0359 (2)	−0.00670 (16)	0.00876 (16)	0.00054 (16)
N1	0.0359 (8)	0.0499 (10)	0.0617 (10)	−0.0109 (7)	0.0234 (7)	0.0004 (8)
N2	0.0414 (9)	0.0472 (10)	0.0674 (11)	−0.0149 (7)	0.0191 (8)	−0.0066 (8)
N3	0.0356 (8)	0.0497 (10)	0.0608 (10)	−0.0139 (7)	0.0207 (7)	−0.0069 (8)
N4	0.0464 (9)	0.0511 (11)	0.0590 (11)	−0.0158 (8)	0.0074 (8)	−0.0075 (8)
N5	0.0626 (12)	0.0949 (16)	0.0629 (12)	−0.0335 (11)	0.0254 (10)	−0.0039 (11)
N6	0.0365 (8)	0.0494 (9)	0.0421 (8)	−0.0128 (7)	0.0095 (6)	−0.0086 (7)
N7	0.0426 (9)	0.0297 (8)	0.0441 (9)	0.0030 (7)	−0.0009 (7)	0.0037 (7)
C12'	0.0426 (9)	0.0297 (8)	0.0441 (9)	0.0030 (7)	−0.0009 (7)	0.0037 (7)
N8	0.0364 (10)	0.0281 (8)	0.0428 (11)	0.000	0.0125 (9)	0.000
O1	0.0388 (7)	0.0499 (8)	0.0595 (8)	−0.0196 (6)	0.0206 (6)	−0.0140 (6)
O2	0.0427 (7)	0.0598 (9)	0.0770 (10)	−0.0165 (7)	0.0337 (7)	−0.0083 (7)
O3	0.0590 (9)	0.0729 (10)	0.0759 (10)	−0.0122 (8)	0.0474 (8)	−0.0169 (8)
O4	0.0412 (7)	0.0626 (9)	0.0434 (7)	−0.0075 (6)	0.0135 (6)	0.0133 (6)
O5	0.0434 (7)	0.0422 (8)	0.0690 (9)	0.0032 (6)	0.0265 (6)	−0.0008 (6)
O6	0.0359 (7)	0.0646 (9)	0.0534 (8)	−0.0200 (6)	0.0060 (6)	−0.0085 (7)

### Geometric parameters (Å, °)

**Table:**

C1—C6	1.397 (2)	C10—H10	0.9300
C1—C2	1.404 (2)	C11—N5	1.302 (3)
C1—C7	1.477 (2)	C11—N6	1.341 (2)
C2—O3	1.348 (2)	C11—H11	0.9300
C2—C3	1.386 (3)	C12—N7	1.318 (2)
C3—C4	1.372 (2)	C12—N8	1.3299 (18)
C3—H3	0.9300	C12—H12	0.9300
C4—C5	1.389 (2)	S1—O5	1.4390 (14)
C4—H4	0.9300	S1—O4	1.4462 (12)
C5—C6	1.382 (2)	S1—O6	1.4542 (12)
C5—S1	1.7683 (16)	N1—N2	1.340 (2)
C6—H6	0.9300	N1—H1A	0.8600
C7—O2	1.230 (2)	N4—N5	1.348 (2)
C7—O1	1.311 (2)	N4—H4A	0.8589
C8—N1	1.313 (2)	N6—H6A	0.8600
C8—N3	1.317 (2)	N7—N7^i^	1.309 (3)
C8—H8	0.9300	N7—H7	0.8600
C9—N2	1.313 (2)	N8—N7'^i^	1.3299 (19)
C9—N3	1.347 (2)	N8—C12^i^	1.3299 (19)
C9—H9	0.9300	O1—H1	0.8298
C10—N4	1.308 (2)	O3—H3A	0.8349
C10—N6	1.313 (2)		
			
C6—C1—C2	118.65 (15)	N5—C11—H11	123.3
C6—C1—C7	121.60 (15)	N6—C11—H11	123.3
C2—C1—C7	119.74 (15)	N7—C12—N8	111.20 (15)
O3—C2—C3	117.57 (16)	N7—C12—H12	124.4
O3—C2—C1	122.33 (16)	N8—C12—H12	124.4
C3—C2—C1	120.10 (15)	O5—S1—O4	112.75 (8)
C4—C3—C2	120.49 (16)	O5—S1—O6	112.42 (9)
C4—C3—H3	119.8	O4—S1—O6	111.42 (8)
C2—C3—H3	119.8	O5—S1—C5	105.83 (8)
C3—C4—C5	120.22 (16)	O4—S1—C5	108.09 (7)
C3—C4—H4	119.9	O6—S1—C5	105.84 (8)
C5—C4—H4	119.9	C8—N1—N2	110.43 (15)
C6—C5—C4	119.95 (15)	C8—N1—H1A	124.8
C6—C5—S1	119.28 (12)	N2—N1—H1A	124.8
C4—C5—S1	120.75 (13)	C9—N2—N1	102.45 (15)
C5—C6—C1	120.57 (15)	C8—N3—C9	102.77 (15)
C5—C6—H6	119.7	C10—N4—N5	110.21 (16)
C1—C6—H6	119.7	C10—N4—H4A	123.0
O2—C7—O1	122.79 (16)	N5—N4—H4A	126.8
O2—C7—C1	121.63 (17)	C11—N5—N4	102.98 (17)
O1—C7—C1	115.58 (14)	C10—N6—C11	104.11 (17)
N1—C8—N3	110.12 (17)	C10—N6—H6A	127.9
N1—C8—H8	124.9	C11—N6—H6A	127.9
N3—C8—H8	124.9	N7^i^—N7—C12	107.15 (10)
N2—C9—N3	114.23 (18)	N7^i^—N7—H7	126.4
N2—C9—H9	122.9	C12—N7—H7	126.4
N3—C9—H9	122.9	N7'^i^—N8—C12	103.29 (18)
N4—C10—N6	109.32 (18)	C12^i^—N8—C12	103.29 (18)
N4—C10—H10	125.3	C7—O1—H1	108.1
N6—C10—H10	125.3	C2—O3—H3A	100.5
N5—C11—N6	113.38 (19)		
			
C6—C1—C2—O3	−178.42 (17)	C4—C5—S1—O5	137.75 (15)
C7—C1—C2—O3	0.5 (3)	C6—C5—S1—O4	80.28 (15)
C6—C1—C2—C3	0.9 (3)	C4—C5—S1—O4	−101.22 (15)
C7—C1—C2—C3	179.84 (17)	C6—C5—S1—O6	−160.25 (13)
O3—C2—C3—C4	178.88 (17)	C4—C5—S1—O6	18.24 (16)
C1—C2—C3—C4	−0.5 (3)	N3—C8—N1—N2	0.2 (2)
C2—C3—C4—C5	−0.2 (3)	N3—C9—N2—N1	−0.2 (2)
C3—C4—C5—C6	0.4 (3)	C8—N1—N2—C9	0.0 (2)
C3—C4—C5—S1	−178.12 (14)	N1—C8—N3—C9	−0.3 (2)
C4—C5—C6—C1	0.1 (3)	N2—C9—N3—C8	0.3 (2)
S1—C5—C6—C1	178.59 (13)	N6—C10—N4—N5	−0.2 (2)
C2—C1—C6—C5	−0.7 (3)	N6—C11—N5—N4	−0.5 (3)
C7—C1—C6—C5	−179.63 (15)	C10—N4—N5—C11	0.4 (3)
C6—C1—C7—O2	176.66 (17)	N4—C10—N6—C11	−0.1 (2)
C2—C1—C7—O2	−2.3 (3)	N5—C11—N6—C10	0.4 (3)
C6—C1—C7—O1	−3.5 (2)	N8—C12—N7—N7^i^	0.1 (2)
C2—C1—C7—O1	177.61 (16)	N7—C12—N8—N7'^i^	−0.03 (10)
C6—C5—S1—O5	−40.75 (15)	N7—C12—N8—C12^i^	−0.03 (10)

Symmetry codes: (i) −*x*+2, *y*, −*z*+3/2.

### Hydrogen-bond geometry (Å, °)

**Table:**

*D*—H···*A*	*D*—H	H···*A*	*D*···*A*	*D*—H···*A*
N1—H1A···O4^ii^	0.86	2.17	2.9231 (18)	145
N1—H1A···O5^iii^	0.86	2.46	2.984 (2)	120
N4—H4A···N2^iv^	0.86	2.09	2.931 (2)	166
N6—H6A···N6^v^	0.86	1.81	2.667 (3)	175
N7—H7···O6	0.86	2.07	2.885 (2)	159
N7'—H7'···O5	0.86	2.50	3.145 (2)	133
N7'—H7'···O6^vi^	0.86	2.12	2.8104 (19)	137
O3—H3A···O2	0.83	1.78	2.577 (2)	159
O1—H1···N3^vi^	0.83	1.85	2.6791 (19)	178
C8—H8···O2^iv^	0.93	2.50	3.110 (2)	123
C9—H9···N5^vii^	0.93	2.62	3.381 (3)	139
C10—H10···O4^iii^	0.93	2.58	3.177 (2)	122
C10—H10···O5^iii^	0.93	2.47	3.278 (2)	145

Symmetry codes: (ii) *x*−1/2, *y*−1/2, *z*; (iii) *x*−1/2, *y*+1/2, *z*; (iv) *x*, *y*+1, *z*; (v) −*x*+1/2, −*y*+1/2, −*z*+1; (vi) *x*, *y*−1, *z*; (vii) −*x*+1, −*y*+1, −*z*+1.
